# 

**Published:** 1883-01

**Authors:** 


ORIGINAL COMMUNICATIONS:
Removal of Stone from the Bladder by "Lithotrity at a Single Sitting." F. N. Otis, M. D. .... 1
Some of the More Common Rachitic Deformities. V. P. Gibney, M. D. .... 4
Garretson's Operation for the Removal of the Coccyx without Disturbance of the Perineum. S. Parker Cottrell, M. D. .... 8
Removal of Parotid Gland. Claude Browning, M. D. .... 10

ORIGINAL TRANSLATIONS AND ABSTRACTS:
A Thirty-seven-Pound Tumor of the Left Supraseual Capsule .... 12
A Rare Case of Spasm of the Voice .... 13
Snake Bite—Rapid Cure by Carbolic Acid .... 13

SELECTIONS:
Introductory Lecture to the Course of Therapeutics .... 15
Pleurisy in Children .... 21
A Class of Pulpless Teeth—Their Treatment .... 28
Typhoid Fever and Salicylic Acid .... 31
Bacteria .... 33
How Consumption may be Prevented .... 35
Faith as an Element of Success in Medicine .... 37
The Antiseptic Treatment of Typhoid Fever .... 38

EDITORIAL:
Salutational .... 40
Introductory .... 40
Hospital Saturday and Sunday .... 42
Baltimore Infectious Diseases Ordinance .... 43
The Recent Typhoid Epidemic in Paris .... 44
Carbolic Acid .... 45
Etiology of Dental Caries .... 47
Save Your Eyes .... 48
Singular Cause of Death .... 48
Questions of Interest to the Dental Specialist .... 49
Editorial Amenities .... 49
Publishers' Notice .... 49

CURRENT NEWS AND OPINION:
The New York Medico-Legal Society .... 51
The Brooklyn Dental Society .... 51
Frozen Food .... 52
An Undescribed Disease of Infants .... 52

BIBLIOGRAPHICAL .... 54
OBITUARY .... 57
MEDICAL PROGRAMME .... 58
POPULAR SCIENCE DEPARTMENT .... 59
VITAL STATISTICS .... 61

